# Prognostic Accuracy of the GRACE Score in Octogenarians and Nonagenarians with Acute Coronary Syndromes

**DOI:** 10.5935/abc.20170175

**Published:** 2018-01

**Authors:** Antonio Mauricio dos Santos Cerqueira Junior, Luisa Gondim dos Santos Pereira, Thiago Menezes Barbosa de Souza, Vitor Calixto de Almeida Correia, Felipe Kalil Beirão Alexandre, Gabriella Sant’Ana Sodré, Jessica Gonzalez Suerdieck, Felipe Ferreira, Marcia Maria Noya Rabelo, Luis Cláudio Lemos Correia

**Affiliations:** 1 Escola Bahiana de Medicina e Saúde Pública, Salvador, BA - Brazil; 2 Hospital São Rafael, Fundação Monte Tabor, Salvador, BA - Brazil

**Keywords:** Acute Coronary Syndrome / mortality, Aged 80 years and over, Prognosis, Risk Assessment, Data Reliability

## Abstract

**Background:**

The GRACE Score was derived and validated from a cohort in which octogenarians and nonagenarians were poorly represented.

**Objective:**

To test the accuracy of the GRACE score in predicting in-hospital mortality of very elderly individuals with acute coronary syndromes (ACS).

**Methods:**

Prospective observational study conducted in the intensive coronary care unit of a tertiary center from September 2011 to August 2016. Patients consecutively admitted due to ACS were selected, and the very elderly group was defined by age ≥ 80 years. The GRACE Score was based on admission data and its accuracy was tested regarding prediction of in-hospital death. Statistical significance was defined by p value < 0,05.

**Results:**

A total of 994 individuals was studied, 57% male, 77% with non-ST elevation myocardial infarction and 173 (17%) very elderly patients. The mean age of the sample was 65 ± 13 years, and the mean age of very elderly patients subgroup was 85 ± 3.7 years. The C-statistics of the GRACE Score in very elderly patients was 0.86 (95% CI = 0.78 - 0.93), with no difference when compared to the value for younger individuals 0.83 (95% CI = 0.75 - 0.91), with p = 0.69. The calibration of the score in very elderly patients was described by χ^2^ test of Hosmer-Lemeshow = 2.2 (p = 0.98), while the remaining patients presented χ^2^ = 9.0 (p = 0.35). Logistic regression analysis for death prediction did not show interaction between GRACE Score and variable of very elderly patients (p = 0.25).

**Conclusion:**

The GRACE Score in very elderly patients is accurate in predicting in-hospital ACS mortality, similarly to younger patients.

## Introduction

Acute coronary syndromes (ACS) are an important cause of in-hospital death in the Western world.^1,2^ Due to the great heterogeneity of clinical and prognostic presentation of ACS, risk stratification is essential so that more aggressive actions can be adopted toward patients at higher risk. In this context, the GRACE Score is the most accurate predictor of hospital death in ACS.^3-6^

However, the derivation and validation of the GRACE Score were conducted in a low representative cohort of octogenarians or nonagenarians.^3,4^ Provided that old age is an important risk indicator, which accumulates aspects of constitutional fragility and higher prevalence of comorbidities, there are reasons to question whether the GRACE Score has modified accuracy in very elderly people.

The present study aimed to test the hypothesis that the GRACE Score has a satisfactory accuracy in predicting in-hospital death when applied to octogenarian and nonagenarian individuals with ACS. The cohort of Prospective Registry of Acute Coronary Syndromes was used in order to answer this question, comparing the discriminatory capacity and calibration of GRACE among individuals aged ≥ 80 years old *versus* < 80 years old.

## Methods

### Sample selection

Patients consecutively admitted to the coronary unit of the tertiary hospital between September 2011 and August 2016, due to suspected ACS (unstable angina and myocardial infarction) were screened for the study. The inclusion criteria were precordial discomfort within 48 hours prior to admission associated with at least one of the following criteria:

1. Positive myocardial necrosis marker, defined by troponin T ≥ 0.01 ug/L or troponin I > 0.034 g/L, which corresponds to values above 99 percent;^7^
2. Ischemic electrocardiographic alteration, consisting of inversion of the T wave (≥ 0.1 mV) or alterations of the ST segment (≥ 0.05 mV); and
3. Previously documented coronary artery disease, defined by a history of myocardial infarction with Q wave or previous angiography demonstrating coronary obstruction ≥ 70%.

The protocol is in compliance with the Declaration of Helsinki, released by the Research Ethic Committee of the institution and all patients evaluated signed the Informed Consent.

### GRACE Score

The clinical data of each patient’s admission in the emergency unit, electrocardiograms performed within the first 6 hours of treatment, troponin T and troponin I dosage in the first 12 hours of treatment and the value of the first plasma creatinine were used to calculate the GRACE Score. The increased myocardial necrosis marker as a component of the scores was defined as troponin over 99 percent. The GRACE Score consists of eight variables: five semi-quantitative ones, *i.e.*, different weight for each age range (systolic blood pressure, heart rate, plasma creatinine and Killip class); and three dichotomic ones (ST segment depression, elevation of myocardial necrosis marker and cardiac arrest at the moment of admission). The final score can range from 0 to 372.^4^

### Data analysis

The accuracy of the GRACE Score was evaluated by discrimination and calibration analyses, which were compared between two groups: one referred to as “very elderly” and the other as “not very elderly”; the first one defined by individuals ≥ 80 years old. The GRACE Score has its performance evaluated by the ability to predict death by any given cause during the hospitalization period.

### Statistical analysis

Numerical variables were expressed as mean and standard deviation when presenting normal distribution or a small deviation from normality, whilst median and interquartile interval were preferable in the presence of at least a moderate deviation from normality. The analysis of normality was performed through combined visualization of the histogram and Q-Q plots, description of *skewness* and *kurtosis* with confidence intervals, and normality tests (Shapiro-Wilk and Kolmogorov-Smirnov). Continuous variables were compared by the Student’s t-test or Wilcoxon test when they presented normal and non-normal distribution, respectively. Categorical variables were expressed in proportion and compared through the χ^2^ test .

The discriminatory capacity of the GRACE Score for mortality was evaluated by the area below the curve of receiver operator characteristics - ROC (statistic-C), which was compared between the two groups by the unpaired Hanley-McNeil test.^8^ The calibration of the scores had a hypothesis test carried out by the Hosmer-Lemeshow technique and was described by the comparison between mortality predicted by GRACE and the one observed in each prediction quartile. The influence of age in the performance of GRACE was tested by the p-value of the interaction by logistic regression analysis.

The SPSS software, version 21, was used. The statistical significance was defined by two-tailed p-value lower than 0.05.

## Results

### Characteristics of the sample

A total of 994 individuals were studied, of which 57% were male and 77% had non-ST elevation ACS. The mean age of the sample was 65 ± 13 years old, of which 173 (17%) were classified as very elderly for being 80 years old or older. The mean age of the very elderly was 85 ± 3.7 years old, compared to 61 ± 11 years of age in the rest of the sample (p < 0.001). The GRACE Score for very elderly patients was 162 ± 34, significantly higher than the one of other patients (115 ± 35; p < 0.001). This higher score in GRACE for very elderly people is due to the difference not only in age, but also in the variables troponin, non-ST elevation, Killip and blood pressure. Percutaneous revascularization during hospitalization was similar in both groups, while surgical revascularization was less frequent in the group of the very elderly. During hospitalization, in-hospital mortality was 5.8% of the total sample, being significantly higher in the group of very elderly people in relation to patients with less than 80 years of age (16% *versus* 3.7%; p < 0.001) (Table 1).

**Table 1:** Comparison of clinical characteristics, laboratory characteristics, GRACE Score and mortality between very elderly versus not very elderly

	Age ≥ 80	Age < 80	p-value
Sample size	173 (17%)	821 (83%)	-
Age (years)	85 ± 3.7	61 ± 11	< 0.001^‡^
Male	82 (47.0%)	487 (59.0%)	0.004^§^
Non-ST elevation ACS	23 (13.0%)	205 (25.0%)	0.001^§^
Diabetes	60 (35.0%)	300 (37.0%)	0.613^§^
Non-ST elevation	55 (32.0%)	308 (37.5%)	0.155^§^
Positive troponin	123 (71.0%)	557 (68.0%)	0.403^§^
**Classification of Killip**			**< 0.001**
Killip I	127 (73.0%)	724 (88.0%)	
Killip II	21 (12.0%)	49 (6.0%)	
Killip III	23 (13.0%)	41 (5.0%)	
Killip IV	2 (1.2%)	7 (0.9%)	
Systolic pressure (mmHg)	151 ± 32	155 ± 30	0.098^‡^
Heart rate	80 ± 17	80 ± 18	0.519^‡^
Serum Creatinine (mg/dl)	1.1 ± 0.5	1.1 ± 0.9	0.669^‡^
Hemoglobin at admission	13 ± 1.8	14 ± 1.9	< 0.001^‡^
Triarterial or LMD*	38 (30.0%)	126 (18.0%)	< 0.001^§^
Percutaneous Coronary Intervention**^†^**	66 (39.0%)	368 (45.0%)	0.129^§^
Revascularization surgery**^†^**	4 (2.0%)	92 (11.0%)	< 0.001^§^
GRACE Score	162 ± 34	115 ± 35	< 0.001^‡^
In-hospital death	28 (16.0%)	30 (4.0%)	< 0.001^§^

ACS: acute coronary syndrome;

* Coronariography performed during hospitalization; LMD: left main disease;

† Myocardial revascularization treatments during hospitalization;

‡ Compared through Student's t-test;

§ Compared through χ^2^ test.

### Discriminatory ability of the GRACE Score

**Figure 1:**
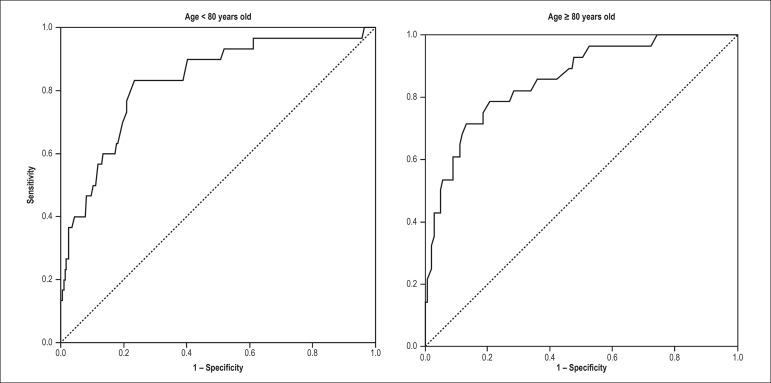
ROC curves of the GRACE Score for the prediction of in-hospital mortality in patients aged ≥ 80 years old versus < 80 years old with acute coronary syndromes. Area below the curve in very elderly was 0.86 (95% CI = 0.78 - 0.93), with no difference in relation to the value found in patients aged < 80 years old (statistic-C = 0.83; 95% CI = 0.75 - 0.91), with p = 0.69 in the comparison between both curves.

In the total sample, the GRACE Score had statistic-C of 0.87 (95% CI = 0.82 - 0.92) in predicting hospital death. GRACE’s statistic-C among the very elderly was 0.86 (95% CI = 0.78 - 0.93), without difference in relation to the value found in patients aged less than 80 years old (statistic-C = 0.83; 95% CI = 0.75 - 0.91), with p = 0.69 in the comparison of both curves (Figure 1). In the logistic regression in which GRACE and very elderly people were simultaneously inserted in the prediction model, there was no interaction between these two variables (p = 0.25). In addition, GRACE remained an independent predictor of age (p < 0.001).

According to the ROC curve, the cutoff score in GRACE with best performance in the group of not very elderly was 134, with sensitivity of 83% and specificity of 76%. Among the very elderly, the cutoff point is displaced upward, with a value of 184, corresponding to the sensitivity of 77% and specificity of 87%.

### Calibration of the GRACE Score

**Figure 2:**
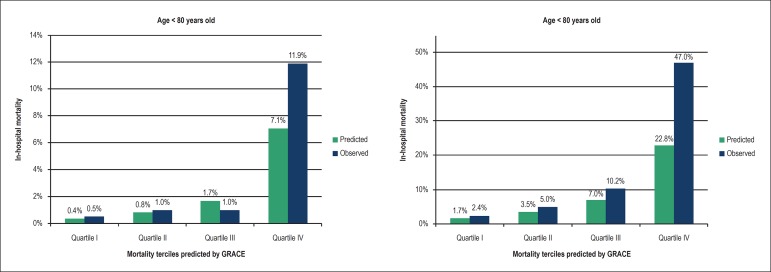
Calibration of the GRACE Score in the prediction of in-hospital mortality in patients aged ≥ 80 years old versus < 80 years old with acute coronary syndromes. The graphics represent the comparison between predicted and observed mortality, in quartiles of probability predicted by the GRACE Score.

In the prediction of the incidence of death during hospitalization, the Hosmer-Lemeshow test showed satisfactory calibration in both groups, very elderly (χ^2^ = 2.2; p = 0.98) and not very elderly (χ^2^ = 9.0; p = 0.35). Figure 2presents the stratified analysis per quartile of the probability predicted by GRACE for hospital death, comparing the predicted and the observed within both age groups. Only the fourth quartile had an underestimated predicted mortality compared to the one observed, in both groups.

## Discussion

The present study demonstrates that the GRACE Score presents satisfactory accuracy in predicting hospital death of very elderly individuals with ACS (octogenarian and nonagenarian ones). The comparison with individuals aged less than 80 years old did not show loss of discriminatory capacity or GRACE’s calibration as the age progressed. Statistic-C values above 0.80 with narrow confidence intervals, in addition to linear growth of mortality observed in the different quartiles of mortality predicted by GRACE, are clear evidence of maintenance of the performance of this score in very elderly. Although the fourth quartile of predicted mortality has underestimated the risk in relation to what was observed, this difference did not compromise the categorization of the fourth larger groups of risk, once that both the observed and the predicted were in mortality ranges considered high for ACS.^4^ There was no interaction between the adequacy of GRACE’s model and the age range group defined by the cutoff point of 80 years of age, confirming GRACE’s accuracy among elderly.

Age is the marker of greater influence on the probability of hospital death in patients hospitalized with ACS, with exponential risk growth as the value of this variable increases.^6,9,10^ The uncertainty of GRACE’s accuracy among very elderly individuals comes from the possibility that there could be less variability of important predicting values within a very advanced age range. For instance, the uniformity of advanced age in this sample may deprive this variable of its discriminatory power, which would not depict great contrast among the individuals. The inclination of this risk function may be lower when there are only very elderly patients. The same may occur with other variables which may be systematically altered in a very elderly sample. Also, the calibration of the score in estimating the numerical risk of death may be different for these patients, once the alpha constant (intercept) tends to be greater in samples with the highest risk. This could explain the need for recalibration of the score.

This uncertainty becomes greater when realized that octogenarian patients were not well represented by the sample which derived and validated the GRACE Score as a hospital death predictor.^4,11,12^ The median age of that sample was 66 years old, with upper limit of 75 years of age for the interquartile interval, indicating that 3/4 of patients were less than 75 years old, with no description as to who were the octogenarian or the nonagenarian ones. Due to the uncertainty of this age range, “very elderly” was defined in our method as people from 80 years of age on, when the occurrence of fragility and comorbidities become more prevalent. Our findings are in agreement with preliminary studies which evaluated the GRACE Score in very elderly, respectively, two European works (Portugal and Spain), and two Chinese ones.^12-15^ Therefore, our results support the literature, being the first to compare the sample of very elderly with individuals aged less than 80 years old. That is, not only do we present an accurate score, but also the suggestion that there is no loss of accuracy.

A risk-treatment paradox depending on age has been described in ACS,^11,12,16-19^ that is, individuals with higher risk being treated in a more conservative way due to the fear of complications, while lower-risk and young individuals receive more aggressive treatment. The use of risk scores in elderly will potentially prevent this paradox, once it allows estimating greater magnitude of the benefit when more aggressive strategies are applied in patients with higher absolute risk derived from GRACE.

On the other hand, it should be recognized that provided the mortality is an outcome resulting from cardiovascular protection *versus* complications in procedures, the greatest benefit in very elderly may be antagonized by greater incidence of complications. Therefore, we emphasize this age range needs validation of the GRACE Score as for the prediction of benefits of more aggressive therapeutic strategies. This is a gap to be filled by future studies.

## Conclusion

In conclusion, the present study represents a favorable evidence to the accurate use of the GRACE Score in the prediction of in-hospital death among octogenarian and nonagenarian patients hospitalized with ACS.
